# Analysis of Nutrition Knowledge After One Year of Intervention in a National Extracurricular Athletics Program: A Cross-Sectional Study with Pair-Matched Controls of Polish Adolescents

**DOI:** 10.3390/nu17010064

**Published:** 2024-12-27

**Authors:** Dominika Skolmowska, Dominika Głąbska, Dominika Guzek, Jakub Grzegorz Adamczyk, Hanna Nałęcz, Blanka Mellová, Katarzyna Żywczyk, Krystyna Gutkowska

**Affiliations:** 1 Department of Dietetics, Institute of Human Nutrition Sciences, Warsaw University of Life Sciences (SGGW-WULS), 159C Nowoursynowska Street, 02-776 Warsaw, Poland; dominika_skolmowska@sggw.edu.pl; 2 Department of Food Market and Consumer Research, Institute of Human Nutrition Sciences, Warsaw University of Life Sciences (SGGW-WULS), 159C Nowoursynowska Street, 02-776 Warsaw, Poland; dominika_guzek@sggw.edu.pl (D.G.); krystyna_gutkowska@sggw.edu.pl (K.G.); 3 Department of Theory of Sport, Józef Piłsudski University of Physical Education, 34 Marymoncka Street, 00-968 Warsaw, Poland; jakub.adamczyk@awf.edu.pl; 4 Pedagogy and Psychology Department, Józef Piłsudski University of Physical Education, 34 Marymoncka Street, 00-968 Warsaw, Poland; hanna.nalecz@awf.edu.pl; 5 Nutrition, Health and Wellness Unit, Nestlé Polska S.A., 32 Domaniewska Street, 02-672 Warsaw, Poland; blanka.mellova@pl.nestle.com (B.M.); katarzyna.zywczyk@pl.nestle.com (K.Ż.)

**Keywords:** nutrition knowledge, consumer nutrition knowledge scale, CoNKS, physical activity, food habits, adolescents

## Abstract

**Background:** Nutrition knowledge may be translated into adequate dietary intake and proper eating habits, so adolescent education programs focusing on improving eating habits and nutrition knowledge are needed. The aim of the cross-sectional study with pair-matched controls was to assess the Consumer Nutrition Knowledge Scale (CoNKS) results and its determinants after one year of intervention in a national extracurricular athletics program within a pair-matched sample of Polish adolescents. **Methods:** The #goathletics Study evaluated a Polish national extracurricular athletics program, ‘Athletics for all’, being a voluntary and free-of-charge physical activity program organized by the Polish Athletics Association. The study allowed comparing the intervention group of adolescents aged 10–14 years, participating in the program for at least 9 months (a school year) and a pair-matched group not participating in it, while the matching was based on city, gender, and age (each group: *n* = 506 adolescents, *n* = 281 females and *n* = 225 males). The nutrition knowledge was assessed using a Consumer Nutrition Knowledge Scale (CoNKS), and during the analysis, the following factors were taken into account: body weight, height, Body Mass Index (BMI), waist circumference, and waist-to-height ratio (WHtR). **Results:** The ‘Athletics for all’ program participation influenced not only the total CoNKS score (*p* < 0.0001) but also the scores in all studied areas—within procedural nutrition knowledge (*p* = 0.0002), declarative nutrition knowledge on nutrients (*p* = 0.0001), and declarative nutrition knowledge on calories (*p* < 0.0001), and program participants revealed a stronger understanding of all the studied areas compared to non-participating individuals. Gender, BMI, and central obesity tendency were not associated with the total CoNKS score (*p* > 0.05), or any of the studied areas (*p* > 0.05). The statistically significant differences in the number of correct answers were observed for four items within procedural nutrition knowledge, for four items within declarative nutrition knowledge on nutrients, and for four items within declarative nutrition knowledge on calories (*p* < 0.05), while for all of them, ‘Athletics for all’ program participants revealed a stronger understanding. **Conclusions:** One year of intervention in a national extracurricular athletics program significantly influenced the nutrition knowledge of the studied group of adolescents aged 10–14 years. While compared with the pair-matched control group of Polish adolescents, they were characterized by a stronger understanding of all areas of nutrition knowledge.

## 1. Introduction

Childhood and early adolescence are critical periods of individuals’ lives, as they pose a transitional time when dietary habits are established, and then they may persist into adulthood [1]. The World Health Organization (WHO) highlights that adolescents are a nutritionally vulnerable group for various specific reasons, including their high requirements due to growth and maturation, their lifestyles and eating patterns, as well as susceptibility to environmental factors [2]. On the one hand, malnutrition is a serious concern in the youth, resulting from inappropriate intake of macro- and micronutrients [3]. On the other hand, a high prevalence of obesity is noted globally among adolescents [4], and according to the World Obesity Federation, it is predicted that the number of obese children and adolescents will rise from 150 million in 2019 to over 250 million by 2030 [5]. In spite of some disparities in the prevalence of childhood obesity-related comorbidities, indicated within a recent systematic review by Obita & Alkhatib [6], it is still emphasized that excessive body mass in this population group is a major healthcare problem.

Childhood obesity results from lifestyle health behaviors associated with socioeconomic factors [7]. However, in order to reduce the risk of excessive body mass in children and adolescents, broadening their nutrition knowledge is necessary, as it is commonly indicated that improved nutrition knowledge is associated with reduced risk of excessive body mass in adolescents [8]. Nutrition knowledge is defined as knowledge of processes and concepts related to nutrition and health, including diet and disease, dietary recommendations, and guidelines [9]. It is indicated that nutrition knowledge may be translated into adequate dietary intake and proper eating habits, while lack of nutrition knowledge is considered one of the main reasons for nutrition-related problems [10].

It is emphasized that adolescents often have insufficient knowledge concerning food and nutrition, including key areas such as nutritional terms, daily nutritional requirements, or choosing healthy snacks [11,12]. Within the study mapping the adolescents’ nutrition knowledge [13], it was indicated that insufficient nutrition knowledge of adolescents is commonly observed independently of the studied country. In the mentioned study, it was proven based on the studies conducted: in Turkey, indicating a very low level of nutrition knowledge in over 65% of the study participants [14]; in the United States, indicating the mean knowledge scores in a group of 7th and 8th graders to be lower than 50% of the possible score to be obtained [15]; in Slovenia, indicating the mean knowledge scores to be only a little bit higher than 50% of the possible score to be obtained [16]; in Iran, indicating correct response rate not higher than 61%, which was observed only for physical education students [17]; and in Poland, indicating insufficient level of nutrition knowledge in over 35% of the study participants [18]. At the same time, it may be indicated that in Polish studies conducted in populations of adolescents, inadequate level of nutrition knowledge was stated in the areas associated with general principles of a healthy diet [19,20], but also in the specific areas associated with fish consumption [21], dietary sources of nutrients [22], or prevention of diet-related diseases [23].

The acquisition of nutrition knowledge depends on a complex interplay of facilitators and barriers that ultimately influence adolescents’ dietary habits [24]. Among such influencing factors are education, income, ethnicity, and gender [24], as females often score higher on nutrition assessment tools than males because they are more concerned about food and health [25].

It is indicated that schools are the most effective venue for organizing health education targeted at dietary changes, where support provided by teachers and parents may bring a positive outcome [26,27], but the nutrition knowledge of adolescents is more important than that of their caregivers to prevent the development of overweight or obesity in a population of adolescents [28]. After-school programs, being an integral part of adolescents’ daily lives, may improve nutrition knowledge and provide proper dietary habits in the youth. A study by Shen et al. [8] confirmed that even a three-month after-school nutrition education program might be beneficial in terms of nutrition knowledge score, as in this study, the dietary habits improved. Similarly, in another school-based interventional study, in which a nutrition education session was conducted, it was stated that after the intervention, there was a significant increase in adolescents’ nutrition knowledge [29].

Taking this into account, and based on the risk of non-communicable diseases resulting from excessive body mass, it is indicated that adolescent education programs focusing on improving eating habits and nutrition knowledge are needed [30,31]. At the same time, such programs should include physical activity education in order to improve physical fitness as well as to influence body mass and to be as effective as possible. Still, it should be emphasized that the effectiveness of such interventions is diverse [32], and it needs to be studied how the effectiveness may be improved.

Based on the presented problems and challenges associated with inadequate nutrition knowledge, the aim of the cross-sectional study with pair-matched controls was to assess the Consumer Nutrition Knowledge Scale (CoNKS) results and its determinants after one year of intervention in a national extracurricular athletics program (‘Athletics for all’, a voluntary and free of charge physical activity program organized by the Polish Athletics Association since 2014) within a pair-matched sample of Polish adolescents.

## 2. Materials and Methods

### 2.1. General Information

The #goathletics Study was a cross-sectional study with pair-matched controls carried out in collaboration of (1) the Institute of Human Nutrition Sciences, Warsaw University of Life Sciences (SGGW-WULS), (2) the Józef Piłsudski University of Physical Education, (3) the Polish Athletics Association, and (4) the Nutrition, Health, and Wellness Unit, Nestlé Polska S.A., as presented previously [33].

The #goathletics Study was developed to evaluate a Polish national extracurricular athletics program, ‘Athletics for all’ (in Polish: Lekkoatletyka Dla Każdego—LDK) [34,35]. ‘Athletics for all’ is a free-of-charge and voluntary extracurricular physical activity program which has been organized by the Polish Athletics Association and has been supported by the Polish Ministry of Sport and Tourism since 2014 [36]. Additionally, Nestlé Polska S.A with the mission to promote a healthy diet, participates in the ‘Athletics for all’ program, while the Institute of Human Nutrition Sciences, Warsaw University of Life Sciences and the Józef Piłsudski University of Physical Education were invited to participate in #goathletics Study (conducted in a sub-population within ‘Athletics for all’ program population) to evaluate the effects of the ‘Athletics for all’ program.

In 2023, over 600 training groups were engaged in the athletics program, regardless of the city size in all the regions of Poland. The program was developed to present a healthy diet as an essential element of physical activity, with nutritional education included in training sessions and presented within the program events. Taking into account that school-based programs may promote fitness, as indicated in Cochrane systematic review by Neil-Sztramko et al. [28], and a healthy diet following, as indicated within a systematic review by Barnes et al. [37], the ‘Athletics for all’ program was developed and introduced in Poland.

The training sessions were conducted by professional trainers by the Polish Athletics Association. The training sessions were conducted based on the same scheme in all training groups, while the common approach was developed and dedicated materials were provided for all the groups, as follows: the basis of the sports training system, scenarios of training sessions, films, and the other visual materials, additional educational materials [38]. Based on the provided materials, the trainers organized the training sessions, taking into account that the core of the ‘Athletics for all’ program is to guarantee regular athletics training (3 sessions/week of 90 min consisting of moderate- to high-intensity physical activity).

The nutritional education was conducted based on the same scheme in all training groups, while the common approach was developed and dedicated materials were provided for all the groups, as follows: the basis of a healthy diet, choice of food products, food products serving sizes, the role of water, the role of breakfast. The developed materials included presentations, scenarios of lessons, activating materials, and e-books for adolescents [39]. The education based on the developed materials was conducted within all the training groups, while it was planned to be conducted within sessions of at least 15 min each, taking into account that the core of the ‘Athletics for all’ program is to guarantee regular athletics training along with nutritional education.

The previous study of the ‘Athletics for all’ program participants revealed that after one year of intervention, lower body mass percentile, Body Mass Index (BMI) percentile, waist circumference (WC), Waist-to-Height Ratio (WHtR), and fat mass share, higher muscle mass share, as well as lower frequency of overweight/obesity and abdominal fat distribution were observed for both boys and girls participating in the physical activity intervention compared to the pair-matched controls [40]. However, the previous study did not assess nutrition knowledge after the intervention, so it was not verified if the observed positive influence should be attributed only to physical activity intervention or also to nutritional education.

The #goathletics Study received approval from the Ethics Committee of the Warsaw University of Life Sciences (09/2023), and all the procedures were in accordance with the Declaration of Helsinki. All participants and their parents/legal guardians provided written informed consent to participate in the study.

### 2.2. Population Studied in #goathletics Study

Within the #goathletics Study, two sub-groups were pair-matched to present the status of the Polish adolescents representing the nationwide sample, as follows:

(1) The intervention group: 506 adolescents (281 female and 225 male, age of 10–14 years), being participants of the ‘Athletics for all’ program for 9 months (in Polish schools, defined as one school year) or longer;
(2) The control group: 506 adolescents (281 female and 225 male, age of 10–14 years), being participants of neither the ‘Athletics for all’ program nor any other physical activity program, for 9 months (in Polish schools, defined as one school year).

Participants of the #goathletics Study were recruited based on the Polish administrative division into voivodeships (16 basic units in Poland) in order to gather a sample from all voivodeships. From each voivodeship, a representative city of medium size was chosen with an ‘Athletics for all’ program conducted within a regular training group. Based on the Polish Central Statistical Office data [41], the sample size for each voivodeship was calculated, taking into account the current data on children aged 10–14 in the voivodeship, in order to maintain the national proportions.

The inclusion and exclusion criteria in terms of the intervention group and control group within the #goathletics Study are presented in Table 1. Due to the fact that within the inclusion criteria for the intervention group, there was regular participation in ‘Athletics for all’ program activities for 9 months, it must be emphasized that the recruitment for the #goathletics Study was conducted within a group already participating in the program (not in a group invited to participate in the program), while the program participation requires regular participation in all its activities (training sessions and nutritional education).

The procedure of pair-matching was based on the variables as follows:

(1) City (pair-matched individual from the intervention group and control group always from the same city);
(2) Gender (pair-matched individual from the intervention group and control group always of the same gender);
(3) Age (pair-matched individuals from the intervention group and control group always with no higher age difference than ±1 year, which was allowed only if it was impossible to conduct matching of identical age individuals).

The variables other than the city, gender, and age were not the basis of the conducted pair-matching, as it was assumed that anthropometric measures, food habits, and physical fitness may be influenced by the ‘Athletics for all’ program participation, so should not be considered in pair-matching. The applied pair-matching method was based on a matched pair case-control model, including the city, gender, and age as pair-matching variables, resulting in an equal number of participants in each group. If more than one possible pair-matching option was observed, the matched individuals were randomly selected, so initially, a higher number of potential control individuals were recruited to obtain exact matching.

Based on the inclusion and exclusion criteria, as well as the recruitment procedure, all the study participants represented a population from all the regions of Poland. The physical activity of all participants from the intervention group and control group resulted from their participation in regular school-based physical education classes (in Poland being attributed to 4 sessions per week of 45 min each [42]) and participation in ‘Athletes for all’ in the intervention group (attributed to 3 sessions per week of 90 min each consisting of moderate- to high-intensity physical activity), while in the control group no any other extracurricular physical activity program or school sports club held at school or any other place for 9 months was allowed. Based on the WHO guidelines on physical activity [43], specifying the recommended physical activity for adolescents as at least 60 min a day, it may be indicated that within the intervention group, over 60 min a day is obtained, so the group presents the recommended physical activity level, which is not obtained in the control group. Neither socioeconomic status nor psychological characteristics were assessed and taken into account for pair-matching.

The geographical distribution in regions of the participants from the intervention group and the control group pair-matched within the study is presented in Table 2.

### 2.3. Anthropometric Measures

Within the #goathletics Study, participants’ body mass was evaluated using Polish OLAF growth reference charts for BMI [44]. In order to measure body mass and height, a weighing scale (calibrated with an accuracy of ±0.1 kg) and a stadiometer (calibrated with an accuracy of ±0.5 cm) were used, and measurement was conducted based on the commonly applied procedure [45]. The WHtR was calculated based on the commonly applied equation [46]. The measured body mass and height values were used to calculate BMI with the use of the Quetelet equation [47]. In order to interpret obtained values, the values of body mass, height, and BMI were compared with standard Polish OLAF growth reference charts, taking into account gender and age [48], in order to define percentile for each study participant while using specific OLAF software available online [44]. The BMI percentile was afterward classified as underweight (BMI < 5th percentile), normal body mass (BMI of 5th–85th percentile), overweight (BMI of 85th–95th percentile), and obesity (BMI > 95th percentile), which was conducted according to growth reference cutoffs by WHO [49].

Independently from the body mass assessment, the tendency for central obesity was assessed based on the waist circumference. For measurement, the mid-abdominal area, in the horizontal plane midway between the lowest ribs and the iliac crest, was chosen based on the commonly applied procedure [50], and the measurement was conducted using a nonelastic flexible measuring tape (calibrated with an accuracy of ±0.5 cm). The measured waist circumference values were compared with the 90th percentile value by gender and age [51], and the waist circumference values were interpreted as follows: central obesity tendency (abdominal fat distribution)—WC ≥ 90th percentile; or no central obesity tendency—WC < 90th percentile [52].

### 2.4. Consumer Nutrition Knowledge Scale (CoNKS)

The nutrition knowledge was assessed using a Consumer Nutrition Knowledge Scale (CoNKS), developed and validated in adults by Dickson-Spillmann et al. [53] as a brief and consumer-oriented tool to assess nutrition knowledge. The validation was conducted in a group of diverse education levels, with the lowest level of primary school completed [53]. This method was indicated by Santos et al. [54] within a group of methods based on the sum of correct answers from a true/false or multiple-choice questionnaire, and it was indicated that this group is recommended to be used in a population of adolescents. Currently, CoNKS is used in large population-based studies, including populations of a low education level [55], as well as it is used in large international studies [56]. Moreover, it is indicated that the statements within the CoNKS are formulated in such a way that simple copying and pasting of a question and searching for an answer would not allow one to find an immediate correct answer [57].

The CoNKS is developed as a list of 20 statements to be assessed as true or false, while they cover the areas of procedural nutrition knowledge (7 statements), declarative nutrition knowledge on nutrients (7 statements), and declarative nutrition knowledge on calories (6 statements) [53]. The CoNKS scale with their correct answers and areas is presented in Table 3.

The CoNKS score ranges from 0 to 20 points. The level of nutrition knowledge of respondents may be divided into 3 categories, based on cutoffs by Bloom [58], as follows: high level of nutrition knowledge for scores of 80–100% (16–20 points), moderate level of nutrition knowledge for scores of 50–79% (10–15 points), and low level of nutrition knowledge for scores of <50% (<10 points).

The percentage of respondents answering correctly for specific statements and the Discrimination Index (DI) were calculated [59]. The DI presents the difference between the share of correct responses for each statement in the upper group (representing the upper 27% of the CoNKS score in the group) and the share of correct responses for each statement in the lower group (representing the lower 27% of the CoNKS score in the group) [60]. The DI ranges from −1.00 to +1.00, and the higher values represent higher discrimination, while the values are interpreted as follows: <0.2—poor discrimination, 0.2–0.29—fair discrimination, 0.3–0.39—good discrimination, ≥0.4—very good discrimination (a high level of effectiveness) [61].

### 2.5. Statistical Analysis

The Shapiro–Wilk test was used to assess the distribution of the data. For groups with the parametric distribution, the *t*-Student’s test and analysis of variance (ANOVA) were used for comparisons. For groups with the non-parametric distribution, the Mann–Whitney U test and Kruskal–Wallis one-way analysis of variance by ranks were used. Additionally, the chi-squared test was applied to compare categorical variables.

A level of *p* ≤ 0.05 was considered statistically significant. All statistical analyses were performed using Statistica 8.0 (Statsoft Inc., Tulsa, OK, USA) and Statgraphics Plus for Windows 4.0 (Statgraphics Technologies Inc., Warrenton, VA, USA).

## 3. Results

The percentage of respondents answering correctly for specific statements and the DI for the statements of the CoNKS in the #goathletics Study in the intervention and control group combined, with statements in the order of increasing DI, is presented in Table 4. The highest percentage of correct responses was observed for the statement “A scoop of chocolate ice cream is just as healthy as a scoop of lemon sorbet” (82.3%), with a good DI of 0.37 (0.3–0.39—good discrimination). A slightly lower share of correct responses was observed for statements associated with energy value, such as “The same amount of sugar and fat contains equally many calories” (81.2%) and “Fat contains fewer calories than the same amount of fiber” (79.3%), while for both sentences, a very good DI of 0.43 and 0.45, respectively, was observed (≥0.4—very good discrimination). The lowest percentage of correct responses was observed for the statement “Brown sugar is much healthier than white sugar” (26.8%), with a good DI of 0.35 (0.3–0.39—good discrimination). In general, for the majority of statements, DI indicated good and very good discrimination, with the highest values (highest level of effectiveness) of ≥0.55 for the statements as follows: “Lentils contain only a few useful nutrients; therefore, their health benefit is not great” (DI = 0.55), “Fat is always bad for your health; you should therefore avoid it as much as possible” (DI = 0.55), and “If you have eaten high-fat foods, you can reverse the effects by eating apples” (DI = 0.56). At the same time, for no statement was the discrimination level defined as fair, and for only three statements was it defined as poor (<0.2), which was observed for the following statements: “Bacon contains more calories than ham” (DI = 0.03), “Pasta with tomato sauce is healthier than pasta with mushroom and cream sauce” (DI = 0.05), and “Oily fish (salmon, mackerel) contain healthier fats than red meat” (DI = 0.07).

The basic characteristics of the participants of the #goathletics Study in the intervention and control group are presented in Table 5. Body weight (in kg and percentiles), BMI (in kg/m^2^ and percentiles), as well as waist circumference, and WHtR were significantly lower in the intervention group compared to the control group (*p* < 0.0001), while there was no difference in age and height (*p* > 0.05). However, the CoNKS score, presenting the level of nutrition knowledge, was significantly higher in the intervention group compared to the control group (*p* < 0.0001).

The basic characteristics of the participants of the #goathletics Study, stratified by CoNKS scores, are presented in Table 6. There was a statistically significant difference in age between the groups, with the oldest participants in the sub-group characterized by a low level of nutrition knowledge (*p* = 0.0404). Body weight and height (in kg/cm and percentiles) did not show statistically significant differences between the sub-groups. A significant difference was found in BMI (in kg/m^2^), with the highest BMI values in the sub-group characterized by the low level of nutrition knowledge (*p* = 0.0332), but it was associated with the age, so while recalculated per percentile, no differences of BMI were observed (*p* > 0.05). However, for waist circumference (*p* = 0.0028) and WHtR (*p* = 0.0015), the highest values were observed in the sub-group characterized by low level of nutrition knowledge, while for moderate and high, the results were comparable.

The CoNKS scores in the areas of procedural nutrition knowledge, declarative nutrition knowledge on nutrients, and declarative nutrition knowledge on calories in sub-groups of the #goathletics Study stratified by intervention are presented in Table 7. The applied intervention (‘Athletics for all’ program participation) was the variable influencing not only the total CoNKS score (*p* < 0.0001) but also the scores in all studied areas—within procedural nutrition knowledge (*p* = 0.0002), declarative nutrition knowledge on nutrients (*p* = 0.0001), and declarative nutrition knowledge on calories (*p* < 0.0001). Namely, ‘Athletics for all’ program participants revealed higher scores across all areas, indicating a stronger understanding of procedural knowledge, nutrient knowledge, and calorie knowledge compared to non-participating individuals.

The CoNKS scores in the areas of procedural nutrition knowledge, declarative nutrition knowledge on nutrients, and declarative nutrition knowledge on calories in sub-groups of the #goathletics Study stratified by gender are presented in Table 8. Gender was the variable associated with neither total CoNKS score (*p* > 0.05) nor any of the studied areas—within procedural nutrition knowledge, declarative nutrition knowledge on nutrients, or declarative nutrition knowledge on calories (*p* > 0.05).

The CoNKS scores in the areas of procedural nutrition knowledge, declarative nutrition knowledge on nutrients, and declarative nutrition knowledge on calories in sub-groups of the #goathletics Study stratified by BMI are presented in Table 9. The BMI was the variable associated with neither total CoNKS score (*p* > 0.05) nor any of the studied areas within procedural nutrition knowledge, declarative nutrition knowledge on nutrients, or declarative nutrition knowledge on calories (*p* > 0.05).

The CoNKS scores in the areas of procedural nutrition knowledge, declarative nutrition knowledge on nutrients, and declarative nutrition knowledge on calories in sub-groups of the #goathletics Study stratified by central obesity tendency are presented in Table 10. The central obesity tendency was the variable not associated with the total CoNKS score (*p* > 0.05) nor any of the studied areas—within procedural nutrition knowledge, declarative nutrition knowledge on nutrients, or declarative nutrition knowledge on calories (*p* > 0.05).

The CoNKS items with the number of correct answers in sub-groups of the #goathletics Study stratified by intervention are presented in Table 11. The statistically significant differences were observed for four items within procedural nutrition knowledge, for four items within declarative nutrition knowledge on nutrients, and for four items within declarative nutrition knowledge on calories (*p* < 0.05), while for all of them, ‘Athletics for all’ program participants revealed a stronger understanding. Among the items with statistically significant differences, there were those of very good discrimination due to DI ≥ 0.4 (9 statements), good discrimination due to DI of 0.3–0.39 (2 statements), but also 1 statement of poor discrimination due to DI < 0.2.

## 4. Discussion

One year of intervention conducted within the ‘Athletics for all’ program turned out to be effective in improving nutrition knowledge in participating adolescents aged 10–14 years. While compared with the pair-matched control group of Polish adolescents, they were characterized by a stronger understanding of all areas of nutrition knowledge, as well as the total CoNKS score. It means that the intervention group improved their nutrition knowledge on such specific topics as nutrients and calories, which may translate into developing healthy food choices and establishing lifelong healthy eating patterns [62]. Interestingly, gender, BMI, and central obesity tendency were not associated with the total CoNKS score or any of the studied areas.

Interventions that focus on the promotion of physical activity as a part of a healthy lifestyle are of great value as they have a direct effect on preventing several chronic diseases, including obesity [63]. It is indicated that various determinants may influence the effectiveness of physical activity interventions and adolescents’ adherence to them, including participants’ ethnicity, socioeconomic status, parental knowledge, or habitual level of physical activity.

In the present study, intervention and control groups comprised Caucasian adolescents, while it was found that ethnicity may be one of the factors potentially influencing the success of lifestyle nutrition and physical activity interventions among children, as the discrepant effectiveness of behavioral interventions in different population groups has been observed [64]. In the systematic review and meta-analysis of Obita & Alkhatib [65], focusing on children from minority ethnic groups, it was found that lifestyle interventions are crucial in case of obesity prevention and management and associated comorbidities in the group of children, but minority ethnicities in high-income countries require more direct lifestyle interventions due to an increased risk of obesity compared to the majority group. Therefore, it is suggested that each physical activity and nutrition intervention should be culturally tailored and include practical nutrition guidance based on affordable/available food products to obtain satisfying results in children and adolescents. Such interventions can be carried out by trainers, health professionals, or physical education teachers, depending on the setting [66].

Another factor that may affect the effectiveness of the applied intervention is socioeconomic status, as indicated in the study of Drenowatz et al. [67], that low socioeconomic status children had lower physical activity levels and exhibited more sedentary behaviors than children from higher income groups. Similar results were obtained by Lee et al. [68], as higher economic status in Korean adolescents favored a higher level of physical activity. Therefore, it may be supposed that adolescents with low socioeconomic status or those with habitual low levels of physical activity may struggle to sufficiently adhere to physical activity interventions. However, as was shown in the study of Alkhatib [69], exercise-based interventions may be successfully conducted even in individuals with a sedentary lifestyle if well-planned, so it may be supposed that the influence of low socioeconomic status may also be counteracted, and the interventions may be effective, but in the presented study it was not verified, as the socio-economic status was not controlled.

Last but not least, parents play a crucial role in the health behaviors of their children [70], and parental knowledge seems to be an important factor in shaping the behaviors of children. In the Kuwaiti study of Allafi et al. [71], there was a positive correlation between the nutritional knowledge of parents and children’s eating habits, and it was summarized that parental knowledge may aid in adopting healthier eating habits in children while understanding the relationship between parents and children in lifestyle risk factors of overweight and obesity is essential for establishing public health policies. Similarly, in the study by Romanos-Nanclares et al. [72], parental nutrition knowledge was associated with adequate intake of various foods, including dairy products, fruit, vegetables, grains, and cereals, in their children. However, in the Polish study by Mazurkiewicz & Raczkowska [73], it was revealed that parental nutrition knowledge does not guarantee implementing theoretical knowledge in the diet of their progeny.

The studies by other authors conducted in other countries also described various interventions and assessed the possibility of influencing the nutrition knowledge of adolescents. The study by Raut et al. [74] in Nepal in a group of students aged 12–19 years indicated that one hour of nutrition education per week, conducted for 12 weeks in the form of mini-lectures and interactive discussions may positively influence not only nutrition knowledge but also attitude. The study conducted by Mancone et al. [75] in Italy in a group of students aged 14–18 years indicated that one hour of nutrition education per week, conducted for 6 weeks, including theoretical learning with practical activities with the integration of digital tools, may positively influence not only nutrition knowledge but also dietary habits. The study conducted by Foo et al. [76] in the United Kingdom in a group of highly trained swimmers that were students aged 14–17 years indicated that half an hour of nutrition education per week, conducted for 7 weeks focusing on sports nutrition, may positively influence sports nutrition knowledge. However, the study conducted by Berger et al. [77] in Israel in a group of students aged about 15 years indicated that two informative lectures regarding nutrition, accompanied by an active sports day and installation in a school facility vending machines containing recommended products, conducted within a school year does not allow to influence nutrition knowledge, even if dietary habits are improved.

The systematic review by Flores-Vázquez et al. [78], assessing educational nutrition interventions conducted in school adolescents and describing 38 various interventions, indicated that within 34 studies, some favorable outcomes were observed, but they were assessed in the areas of not only nutrition knowledge, but also dietary habits, physical activity, lifestyle, and nutritional status. For the nutrition knowledge, it was emphasized that it is necessary to apply techniques that allow to incorporate the novel knowledge [46], which results from the fact that nutrition knowledge is necessary to change nutrition behaviors, even if it is not a sufficient factor to observe change [79].

Importantly, the school-based interventions, as the one presented in the conducted study, are indicated among the most promising approaches, as indicated in the review of the evidence-based nutrition interventions conducted in Indonesia [80]. It was confirmed within the umbrella review by O’Brien et al. [81], describing school-based nutrition interventions conducted in children aged 6–18 years, which concluded that school-based nutrition interventions, including nutrition education, food environment, and other elements, may positively affect some dietary habits. However, in the mentioned umbrella review, the studies were included only if they assessed not only nutrition knowledge but also other outcomes, such as dietary habits or body mass [81].

Taking this into account, it must be indicated that in the studied group, the previous analysis, which included food habits and body mass [33], revealed that regularly participating in the ‘Athletics for all’ program for at least 9 months influenced body weight and BMI, but it did not influence the results obtained while using Adolescents’ Food Habits Checklist (AFHC). Based on the presented results, it may be indicated that even if the changes in food habits are not noticed (which may result not only from lack of such changes but also from the applied tool), after one school year of intervention, physical activity is higher and nutrition knowledge is better in the intervention group than in the control group.

Similar observations are formulated by other authors, noticing that a higher level of nutrition knowledge is not associated with more beneficial food habits [82]. It was also indicated within a systematic review describing the association between nutrition knowledge and dietary behaviors by Thakur & Mathur [83], which indicated in only two studies a statistically significant association between overall nutrition knowledge and practices and concluded that there is a need for holistic behavior change strategies including not only nutrition knowledge but also supporting food skills, food shopping and cooking to encourage healthy eating habits. It indicates two major goals within the nutritional education related to nutrition knowledge, namely, effective transfer of nutrition knowledge as the first one and learning how to transform nutrition knowledge into modified food habits as the second one.

In spite of the fact that the conducted study allows the formulation of important observations associated with the effect of the applied one year of intervention in a national extracurricular athletics program on nutrition knowledge, some limitations of the study should be listed. It should be indicated that the baseline nutrition knowledge (before the ‘Athletics for all’ program participation) was not assessed, but it was compared between the intervention group and the control group. Moreover, the study was conducted in a homogeneous group of adolescents, taking into account their age, education level, and ethnicity, so the assessment of the influence of those socio-demographic features on nutrition knowledge was not possible within the studied sample, which may also be important, as for minority ethnicities obesity prevention requires different perspective [84].

On the other hand, it must be indicated that conducting the study in a large homogeneous sample is a strength of the study, as it allows to generalize during concluding for typical Polish adolescents. The other strengths that must be indicated are conducting a study in a population-based sample from all the regions and all voivodeships of Poland, comparing it with pair-matched controls, and using a validated consumer-oriented tool to assess nutrition knowledge.

## 5. Conclusions

The one year of intervention in a national extracurricular athletics program significantly influenced the nutrition knowledge of the studied group of adolescents aged 10–14 years. Compared with the pair-matched control group of Polish adolescents, they were characterized by a stronger understanding of procedural nutrition knowledge, declarative nutrition knowledge on nutrients, and declarative nutrition knowledge on calories. Gender, BMI, and central obesity tendency were not associated with nutrition knowledge in the studied group. Based on the obtained results, it may be indicated that promoting health education targeted at dietary changes by specialists, with the simultaneous support provided by teachers and parents, may bring a positive outcome for the youth. Taking this into account, effective, complex, and tailored intervention programs aimed at improving adolescents’ nutrition knowledge are currently needed.

## Figures and Tables

**Table 1:** The inclusion and exclusion criteria of the intervention group and control group within #goathletics Study.

**Inclusion Criteria**	
**Intervention Group**	**Control Group**
Caucasian	Caucasian
Age of 10–14	Age of 10–14
Comparable share of female and male adolescents	Comparable share of female and male adolescents
Written informed consent for participation in #goathletics Study	Written informed consent for participation in #goathletics Study
Written informed consent from parents for their child to participate in the #goathletics Study	Written informed consent from parents for their child to participate in the #goathletics Study
Participating in the ‘Athletics for all’ program within a regular training group (1 regular training group chosen for each voivodeship in a representative city of medium size)	Students from a school with the ‘Athletics for all’ program never introduced
Regular participation in ‘Athletics for all’ program activities for 9 months (in Polish schools, defined as one school year) or longer (at least from September 2022 to June 2023)	Participants of neither the ‘Athletics for all’ program nor any other extracurricular physical activity program or school sports club held at school or any other place for 9 months (in Polish schools, defined as one school year) or longer (at least from September 2022 to June 2023)—verified based on both parents and children declarations
-	Pair-matching with participants from the intervention group
**Exclusion Criteria**	
**Intervention Group**	**Control Group**
Diagnosis of motor dysfunctions	Diagnosis of motor dysfunctions
Diagnosis of mental disability	Diagnosis of mental disability
Any missing data	Any missing data
Taking part in another physical activity program other than regular school-based physical education classes.	-

**Table 2:** The geographical distribution in regions of the participants from the intervention group and the control group pair-matched within the study.

Region of Poland	Gender	Intervention Group	Control Group
Central	Male	*n* = 23	*n* = 23
	Female	*n* = 22	*n* = 22
Mazovia	Male	*n* = 41	*n* = 41
	Female	*n* = 35	*n* = 35
South-west	Male	*n* = 23	*n* = 23
	Female	*n* = 23	*n* = 23
South	Male	*n* = 52	*n* = 52
	Female	*n* = 52	*n* = 52
North-west	Male	*n* = 28	*n* = 28
	Female	*n* = 56	*n* = 56
North	Male	*n* = 25	*n* = 25
	Female	*n* = 55	*n* = 55
East	Male	*n* = 33	*n* = 33
	Female	*n* = 38	*n* = 38

**Table 3:** The Consumer Nutrition Knowledge Scale (CoNKS) items with their correct answer.

Items	Number of Question	True/ False
**Procedural nutrition knowledge (0–7 points)**		
If you have eaten high-fat foods, you can reverse the effects by eating apples	2	F
A healthy meal should consist of half meat, a quarter vegetables, and a quarter side dishes	4	F
Fat is always bad for your health; you should, therefore, avoid it as much as possible	7	F
A balanced diet implies eating all foods in the same amounts	9	F
For a healthy nutrition, dairy products should be consumed in the same amounts as fruit and vegetables	18	F
Brown sugar is much healthier than white sugar	20	F
To eat healthily, you should eat less fat. Whether you also eat more fruit and vegetables does not matter	13	F
**Declarative nutrition knowledge on nutrients (0–7 points)**		
Oily fish (salmon, mackerel) contain healthier fats than red meat	12	T
Lentils contain only a few useful nutrients; therefore, their health benefit is not great	1	F
A salad dressing made with mayonnaise is as healthy as the same dressing made with mustard	6	F
Pasta with tomato sauce is healthier than pasta with mushroom and cream sauce	8	T
Skimmed milk contains fewer minerals than full-fat milk	19	F
The health benefit of fruit and vegetables lies alone in the supply of vitamins and minerals	10	F
A scoop of chocolate ice cream is just as healthy as a scoop of lemon sorbet	14	F
**Declarative nutrition knowledge on calories (0–6 points)**		
If cream is whipped, it contains fewer calories than in its liquid form	3	F
Bacon contains more calories than ham	11	T
Fat contains fewer calories than the same amount of fiber	5	F
The same amount of beef steak and chicken breast contains equally many calories	15	F
A sandwich with mozzarella contains as many calories as the same sandwich with Emmental/Swiss cheese	17	F
The same amount of sugar and fat contains equally many calories	16	F

**Table 4:** The percentage of respondents answering correctly for specific statements and the Discrimination Index (DI) for the statements of the Consumer Nutrition Knowledge Scale (CoNKS) in the #goathletics Study in the intervention and control group combined (*n* = 1012) (statements in the order of increasing DI).

Items	% of Participants with	
	Discrimination Index	Correct Answer
Bacon contains more calories than ham	0.03	71.6
Pasta with tomato sauce is healthier than pasta with mushroom and cream sauce	0.05	56.1
Oily fish (salmon, mackerel) contain healthier fats than red meat	0.07	63.2
Brown sugar is much healthier than white sugar	0.35	26.8
A scoop of chocolate ice cream is just as healthy as a scoop of lemon sorbet	0.37	82.3
A sandwich with mozzarella contains as many calories as the same sandwich with Emmental/Swiss cheese	0.42	73.4
The same amount of sugar and fat contains equally many calories	0.43	81.2
To eat healthily, you should eat less fat. Whether you also eat more fruit and vegetables does not matter	0.44	64.5
Fat contains fewer calories than the same amount of fiber	0.45	79.3
For a healthy nutrition, dairy products should be consumed in the same amounts as fruit and vegetables	0.45	72.2
The same amount of beef steak and chicken breast contains equally many calories	0.47	78.6
If cream is whipped, it contains fewer calories than in its liquid form	0.48	79.0
A salad dressing made with mayonnaise is as healthy as the same dressing made with mustard	0.48	73.9
A balanced diet implies eating all foods in the same amounts	0.49	73.6
The health benefit of fruit and vegetables lies alone in the supply of vitamins and minerals	0.50	58.7
Skimmed milk contains fewer minerals than full-fat milk	0.51	51.8
Lentils contain only a few useful nutrients; therefore, their health benefit is not great	0.55	72.6
Fat is always bad for your health; you should, therefore, avoid it as much as possible	0.55	61.9
If you have eaten high-fat foods, you can reverse the effects by eating apples	0.56	74.9

**Table 5:** The basic characteristics of the participants of the #goathletics Study in the intervention (*n* = 506) and control group (*n* = 506).

Characteristics	Total (*n* = 1012)		Intervention Group (*n* = 506)		Control Group (*n* = 506)		***p***
	Mean (SD)	Median (Min–Max)	Mean (SD)	Median (Min–Max)	Mean (SD)	**Median (Min–Max)**	
Age (year)	12.22 (1.06)	12 * (10–14)	12.23 (1.09)	12 * (10–14)	12.21 (1.03)	12 * (10–14)	0.8539
Body weight (kg)	45.08 (10.27)	44 * (23–92)	43.6 (9.21)	43 * (24.6–88.8)	46.56 (11.04)	45 * (23–92)	<0.0001 **
Body weight (percentile)	49.44 (27.6)	49 * (0–137)	45.34 (25.47)	44.5 * (1–99)	53.53 (29.04)	55 * (0–137)	<0.0001 **
Height (cm)	155.02 (9.4)	155 * (126.5–188.5)	155.42 (9.63)	155 (126.5–188.5)	154.62 (9.16)	154.5 (130–186)	0.1710
Height (percentile)	51.33 (29.46)	52 * (0–100)	52.5 (28.86)	54 * (0–100)	50.16 (30.02)	51 * (0–100)	0.2037 **
BMI (kg/m^2^)	18.58 (2.94)	18.11 * (12.94–35)	17.88 (2.26)	17.65 * (12.94–28.34)	19.29 (3.34)	18.79 * (13–35)	<0.0001 **
BMI (percentile)	48.64 (27.56)	48 * (0–100)	42.47 (24.94)	41 * (0–98)	54.81 (28.68)	58 * (1–100)	<0.0001 **
Waist circumference (cm)	66.28 (8.74)	65 * (29.67–100.17)	64.82 (7.74)	64 * (45–96.33)	67.73 (9.44)	66 * (29.67–100.17)	<0.0001 **
WHtR (-)	0.43 (0.05)	0.42 * (0.2–0.62)	0.42 (0.04)	0.41 * (0.31–0.56)	0.44 (0.06)	0.43 * (0.2–0.62)	<0.0001 **
CoNKS score (-)	13.48 (3.34)	14 * (3–20)	14.07 (3.08)	15 * (4–20)	12.89 (3.47)	13 * (3–20)	<0.0001 **

* non-parametric distribution (*p* < 0.05; Shapiro–Wilk test); ** Mann–Whitney U test (for non-parametric distribution), for other—*t*-Student’s test; BMI—Body Mass Index; WHtR—Waist-to-Height Ratio; CoNKS—Consumer Nutrition Knowledge Scale.

**Table 6:** The basic characteristics of the participants of the #goathletics Study, stratified by Consumer Nutrition Knowledge Scale (CoNKS) scores (*n* = 1012).

Characteristics	High Level of Nutrition Knowledge (*n* = 314)		Moderate Level of Nutrition Knowledge (*n* = 561)		Low Level of Nutrition Knowledge (*n* = 137)		*p* **
	Mean (SD)	Median (Min–Max)	Mean (SD)	Median (Min–Max)	Mean (SD)	**Median (Min–Max)**	
Age (year)	12.27 (1.04)	12 (10–14) *^ab^	12.15 (1.09)	12 (10–14) *^a^	12.39 (0.93)	12 (10–14) *^b^	0.0404
Body weight (kg)	45.3 (11.21)	44 (23–88.8) *	44.64 (9.88)	43.6 (23–92) *	46.38 (9.5)	45.2 (27–88) *	0.0759
Body weight (percentile)	48.36 (28.84)	48 (0–99) *	49.29 (27.15)	47 (0–100) *	52.53 (26.49)	55 (1–137) *	0.3491
Height (cm)	155.67 (9.64)	155 (126.5–188.5) *	154.48 (9.33)	155 (130–186)	155.71 (9.05)	155 (136–181)	0.2253
Height (percentile)	52.31 (29.75)	54 (0–100) *	51.35 (29.3)	52 (0–100) *	49.02 (29.48)	49 (0–100) *	0.5398
BMI (kg/m^2^)	18.49 (3.21)	17.89 (12.94–35) *^a^	18.54 (2.83)	18.08 (13.32–33.79) *^ab^	18.99 (2.7)	18.9 (13.78–29.75) *^b^	0.0332
BMI (percentile)	46.69 (28.97)	44.5 (0–100) *	48.65 (27.02)	48 (0–100) *	53.05 (26.11)	57 (1–99) *	0.0738
Waist circumference (cm)	65.67 (8.89)	64.33 (44.33–96.33) *^a^	66.12 (8.73)	65 (29.67–100.17) *^a^	68.29 (8.24)	66 (52.67–96.33) *^b^	0.0028
WHtR (-)	0.42 (0.05)	0.41 (0.27–0.62) *^a^	0.43 (0.05)	0.42 (0.20–0.62) *^a^	0.44 (0.05)	0.43 (0.35–0.62) *^b^	0.0015
CoNKS score (-)	17.16 (1.08)	17 (16–20) *^a^	12.83 (1.67)	13 (10–15) *^b^	7.72 (1.48)	8 (3–9) *^c^	<0.0001

* non-parametric distribution (*p* < 0.05; Shapiro–Wilk test); ** Kruskal–Wallis ANOVA test; BMI—Body Mass Index; WHtR—Waist-to-Height Ratio; ^a, b, c^—different letters in rows indicate statistically significant differences.

**Table 7:** The Consumer Nutrition Knowledge Scale (CoNKS) scores in the areas of procedural nutrition knowledge, declarative nutrition knowledge on nutrients, and declarative nutrition knowledge on calories in sub-groups of the #goathletics Study stratified by intervention (*n* = 1012).

	Intervention Group (*n* = 506)		Control Group (*n* = 506)		*p* **
	Mean (SD)	Median (Min–Max)	Mean (SD)	Median (Min–Max)	
CoNKS total	14.07 (3.08)	15 * (4–20)	12.89 (3.47)	13 * (3–20)	<0.0001
Procedural nutrition knowledge score	4.47 (1.65)	5 * (0–7)	4.05 (1.76)	4 * (0–7)	0.0002
Declarative nutrition knowledge on nutrient score	4.77 (1.31)	5 * (0–7)	4.41 (1.47)	5 * (0–7)	0.0001
Declarative nutrition knowledge on calorie score	4.84 (1.18)	5 * (1–6)	4.43 (1.34)	5 * (1–6)	<0.0001

* non-parametric distribution (*p* < 0.05; Shapiro–Wilk test); ** Mann–Whitney U-test.

**Table 8:** The Consumer Nutrition Knowledge Scale (CoNKS) scores in the areas of procedural nutrition knowledge, declarative nutrition knowledge on nutrients, and declarative nutrition knowledge on calories in sub-groups of the #goathletics Study stratified by gender (*n* = 1012).

	Girls (*n* = 562)		Boys (*n* = 450)		*p* **
	Mean (SD)	Median (Min–Max)	Mean (SD)	Median (Min–Max)	
CoNKS total	13.55 (3.34)	14 * (3–20)	13.39 (3.33)	14 * (4–20)	0.4782
Procedural nutrition knowledge score	4.25 (1.74)	4 * (0–7)	4.28 (1.69)	4 * (1–7)	0.8866
Declarative nutrition knowledge on nutrients score	4.63 (1.36)	5 * (0–7)	4.53 (1.45)	5 * (0–7)	0.3680
Declarative nutrition knowledge on calories score	4.67 (1.3)	5 * (1–6)	4.58 (1.24)	5 * (1–6)	0.1292

* non-parametric distribution (*p* < 0.05; Shapiro–Wilk test); ** Mann–Whitney U-test.

**Table 9:** The Consumer Nutrition Knowledge Scale (CoNKS) scores in the areas of procedural nutrition knowledge, declarative nutrition knowledge on nutrients, and declarative nutrition knowledge on calories in sub-groups of the #goathletics Study stratified by Body Mass Index (BMI) (*n* = 1012).

	Underweight (*n* = 42)		Normal Weight (*n* = 845)		Overweight (*n* = 85)		Obesity (*n* = 40)		*p* **
	Mean (SD)	Median (Min–Max)	Mean (SD)	Median (Min–Max)	Mean (SD)	Median (Min–Max)	Mean (SD)	Median (Min–Max)	
CoNKS total	14.6 (3.48)	15.5 (6–20)	13.42 (3.34)	14 * (3–20)	4.6 (1.28)	5 (1–6)	13.9 (2.99)	14 (6–19)	0.1246
Procedural nutrition knowledge score	4.55 (1.99)	5 * (1–7)	4.26 (1.7)	4 * (0–7)	13.33 (3.33)	14 * (6–19)	4.58 (1.39)	5 * (2–7)	0.2146
Declarative nutrition knowledge on nutrient score	4.98 (1.51)	5 * (2–7)	4.56 (1.41)	5 * (0–7)	4 (1.86)	4 * (1–7)	4.58 (1.32)	5 * (1–7)	0.3074
Declarative nutrition knowledge on calorie score	5.07 (1.02)	5 * (2–6)	4.6 (1.28)	5 * (1–6)	4.71 (1.29)	5 * (2–7)	4.75 (1.26)	5 * (1–6)	0.1269

* non-parametric distribution (*p* < 0.05; Shapiro–Wilk test); ** Mann–Whitney U-test.

**Table 10:** The Consumer Nutrition Knowledge Scale (CoNKS) scores in the areas of procedural nutrition knowledge, declarative nutrition knowledge on nutrients, and declarative nutrition knowledge on calories in sub-groups of the #goathletics Study stratified by central obesity tendency (*n* = 1012).

	No Central Obesity Tendency (*n* = 865)		Tendency for Central Obesity (*n* = 147)		*p* **
	Mean (SD)	Median (Min–Max)	Mean (SD)	Median (Min–Max)	
CoNKS total	13.50 (3.35)	14 * (3–20)	13.36 (3.27)	14 (5–20)	0.6000
Procedural nutrition knowledge score	4.27 (1.73)	4 * (0–7)	4.22 (1.66)	4 * (1–7)	0.7684
Declarative nutrition knowledge on nutrient score	4.60 (1.42)	5 * (0–7)	4.51 (1.29)	5 * (1–7)	0.4289
Declarative nutrition knowledge on calorie score	4.63 (1.26)	5 * (1–6)	4.63 (1.37)	5 * (1–6)	0.7392

* non-parametric distribution (*p* < 0.05; Shapiro–Wilk test); ** Mann–Whitney U-test.

**Table 11:** The Consumer Nutrition Knowledge Scale (CoNKS) items with the number of correct answers in sub-groups of the #goathletics Study stratified by intervention (*n* = 1012).

Items	Correct Answers		*p*
	Intervention Group (*n* = 506)	Control Group (*n* = 506)	
**Procedural nutrition knowledge items**			
If you have eaten high-fat foods, you can reverse the effects by eating apples	405 (80.0%)	353 (69.8%)	0.0002
A healthy meal should consist of half meat, a quarter vegetables and a quarter side dishes	265 (52.4%)	262 (51.8%)	0.9982
Fat is always bad for your health; you should therefore avoid it as much as possible	338 (66.8%)	288 (56.9%)	0.0015
A balanced diet implies eating all foods in the same amounts	394 (77.9%)	351 (69.4%)	0.0027
For a healthy nutrition, dairy products should be consumed in the same amounts as fruit and vegetables	367 (72.5%)	364 (71.9%)	0.9976
Brown sugar is much healthier than white sugar	152 (30.0%)	119 (23.5%)	0.0231
To eat healthily, you should eat less fat. Whether you also eat more fruit and vegetables does not matter	340 (67.2%)	313 (61.9%)	0.3692
**Declarative nutrition knowledge on nutrients items**			
Oily fish (salmon, mackerel) contain healthier fats than red meat	333 (65.8%)	307 (60.7%)	0.4113
Lentils contain only few useful nutrients, therefore their health benefit is not great	382 (75.5%)	353 (69.8%)	0.0484
A salad dressing made with mayonnaise is as healthy as the same dressing made with mustard	400 (79.1%)	348 (68.8%)	0.0003
Pasta with tomato sauce is healthier than pasta with mushroom and cream sauce	303 (59.9%)	265 (52.4%)	0.0191
Skimmed milk contains fewer minerals than full-fat milk	269 (53.2%)	255 (50.4%)	0.2421
The health benefit of fruit and vegetables lies alone in the supply of vitamins and minerals	296 (58.5%)	298 (58.9%)	0.9995
A scoop of chocolate ice cream is just as healthy as a scoop of lemon sorbet	429 (84.8%)	404 (79.8%)	0.0480
**Declarative nutrition knowledge on calories items**			
If cream is whipped it contains less calories than in its liquid form	414 (81.8%)	385 (76.1%)	0.0308
Bacon contains more calories than ham	375 (74.1%)	350 (69.2%)	0.3851
Fat contains fewer calories than the same amount of fiber	425 (84.0%)	378 (74.7%)	0.0004
The same amount of beef steak and chicken breast contains equally many calories	422 (83.4%)	373 (73.7%)	0.0002
A sandwich with mozzarella contains as many calories as the same sandwich with Emmental/Swiss cheese	381 (75.3%)	362 (71.5%)	0.6086
The same amount of sugar and fat contains equally many calories	430 (85.0%)	392 (77.5%)	0.0029

## Data Availability

The data presented in this study can be obtained upon request from the corresponding author. Due to ethical restrictions, the data are not publicly available.
